# Learner engagement in the flipped foreign language classroom: Definitions, debates, and directions of future research

**DOI:** 10.3389/fpsyg.2022.810701

**Published:** 2022-07-14

**Authors:** Zhiyong Li, Jiaying Li

**Affiliations:** ^1^Public Language Education Department, Nankai University Binhai College, Tianjin, China; ^2^University Libraries, Nankai University, Tianjin, China

**Keywords:** flipped classrooms, learner engagement, engagement construct, EFL context, learner dynamics

## Abstract

Flipped classrooms have attracted widespread attention and interest from English for foreign language (EFL) practitioners and researchers and are regarded as a promising pedagogical approach to increase learning outcomes and facilitate learner engagement. This article takes stock of the publications on learner engagement in flipped EFL classrooms. In so doing, we aim to clarify the engagement construct specific to the EFL classroom setting, summarize the effect that flipped classrooms have on EFL learners’ engagement, and identify factors affecting engagement. We find that there has been a range of debates and tensions regarding flipped classrooms and learner engagement in EFL settings, and that more theory-grounded empirical studies are needed to delineate learner engagement in localized flipped learning and teaching contexts. We would argue that in future research, multiple variables, including learner dynamics and social-psychological factors, should be considered while flipping the EFL course so that practitioners can provide tailor-made support to improve learners’ engagement levels.

## Introduction

Since entering the 21st century, technology has played an increasingly important role in facilitating learners’ motivation and engagement in the language classroom. In fact, with the advent of computers and the Internet, language learning has entered a new phase of development, marked by practicing the language on social networking websites, acquiring grammar and vocabulary through mobile phone apps, and attending virtual training delivered by lecturers living at the other end of the world (Chang and Hung, 2019). However, the near-ubiquity of digital devices also means that it is imperative for language teachers to know how to integrate modern technologies into their classrooms to create an engaging environment tailored to the needs of learners in the face of distractions caused by digital gadgets (Li, 2021).

Given the popularity of learner-centered approach and the widespread applications of modern technologies to reach this goal, there has been a growing body of research on the impact of the technology-assisted teaching approach on learners’ engagement. Against this background, the flipped classrooms have garnered increasing attention (Hung, 2015; Bond, 2020), especially after Bergmann and Sams (2012) promoted the flipped classroom in the educational area. However, despite the popularity of flipped classrooms and researchers’ attempts to delineate learner engagement, there is limited effort to synthesize learner engagement in different flipped EFL settings. EFL refers to the environment where English is neither widely spoken in daily communications nor is it a medium of instruction in educational settings (Carter and Nunan, 2000). Moreover, learner engagement is a multifaceted, multidimensional, and dynamic construct dependent on the unique context of learning and teaching. Therefore, it is necessary to gain a clear comprehension of learner engagement in various flipped EFL contexts.

## The definition of flipped classrooms

As previously mentioned, the flipped classroom have enjoyed popularity because of their communicative-based, learner-centered approach to teaching and learning (Wanner and Palmer, 2015). Its definition, however, varies in different situations. Sometimes it refers to the blended classroom (Lage and Platt, 2000), in which traditional teaching is mixed with learner-centered in-class discussions and pre-learning materials. Sometimes it shares the name with just-in-time teaching (Novak, 2011), through which teachers prepare pre-class instructional materials to stimulate learners’ interest so that they can engage in the classroom learning tasks. Bergmann and Sams (2012) call it the flipped classroom based on their success story of giving students access to various short videos uploaded on the Internet before students attend the class.

Bishop and Verleger (2013) define the flipped classroom by emphasizing that a flipped classroom should only include computer-based pre-class individual instructions to prepare students for interactive-based group learning activities inside the classroom. In other words, they exclude test-based materials and non-video sources that can prepare students before the class. On the other hand, the Flipped Learning Network (FLN) (2014) define flipped classrooms as an instructional approach through which teachers create videos or use videos from other sources for students to access before the class; in this way, instructors can adopt student-centered, active learning strategies for learners to integrate and apply the knowledge in the course. In the flipped classroom, teachers act as guides and facilitators who provide individualized student support. The definitions provided by Bishop and Verleger and the Flipped Learning Network seem to stress the indispensability of videos for implementing flipped classrooms. However, it remains unclear whether only videos qualify for pre-class materials. As Bond (2020) and Lo and Hew (2021) claim, there is still debate over what constitutes a flipped classroom. However, the essence of the pedagogy is learners’ preparation before class and the subsequent group learning space for interactive-based activities during the class. Thus, despite different ways of defining flipped classrooms, educational researchers tend to agree that flipped classrooms involve the use of technology to support collaborative-based learning and that the flipped approach puts students at the center, with the class design and learning materials revolving around how to address learners’ needs and arouse learner engagement.

## The definition of learner engagement

Engagement is a crucial topic in education and has been increasingly studied and discussed in the academic field. Finn (1989) categorized student engagement as participation and identification. Participation refers to students’ learning behaviors in the school, and identification is related to affective engagement, such as whether students have developed a sense of belonging to the learning community and whether they have a positive relationship with the school and staff. However, Finn’s definition of engagement construct is confined to only behavioral and affective levels. Engagement may go beyond our observations and be understood from psychological and social perspectives (Kahu, 2013). Fredricks et al. (2004) provide a more comprehensive categorization of student engagement, and they divide engagement into behavioral, affective, and cognitive engagement. Behavioral engagement can be understood by studying how students behave in the classroom and at school and whether they are actively involved in learning tasks and school activities. Affective engagement refers to student emotions such as happiness, sadness, boredom, anxiety, and interest displayed in learning. Finally, cognitive engagement is defined as students’ mental effort in their learning and self-reflection of their learning strategies (Fredricks et al., 2004).

## Learner engagement in language classrooms

Engagement also has become a prominent area of inquiry in second language acquisition (Hiver et al., 2020). The foci of engagement, which are related to active involvement and sustained participation, are compatible with the nature of second language acquisition. This is because acquiring a language takes time and requires sustained effort over an extended period (Mercer and Dörnyei, 2020). Also, a second language can be more effectively acquired when the learners are situated in a communicative environment. This means high-level learner engagement can ensure persistent devotion of time and energy from language learners. Therefore, learner engagement in the EFL context has significant implications for EFL learners, teaching professionals and researchers.

In language education, Bygate and Samuda (2009) define engagement as the learners’ efforts and resources to achieve learning objectives. Svalberg (2018) approaches engagement from the learners’ language awareness perspective. Under the framework of engagement with language, Svalberg (2009, 2018) maintains that behavioral engagement in the language setting refers to the time, energy, and quality of participation in the language learning tasks; cognitive engagement refers to the level of learners’ attention on the learning tasks, their reflections over the success and failures while completing language tasks, and self-regulating behaviors and problem-solving abilities in the language classroom; the affective engagement in language tasks can be delineated as positive emotions (interest, enthusiasm, and curiosity) and negative emotions or disengagement (boredom, frustration, and anxiety) in the language classroom. Finally, social engagement refers to participants cooperating and interacting to complete language tasks.

Mercer and Dörnyei (2020) open up a new dimension of studying learner engagement in language classrooms by drawing on the complex dynamic systems theory. They argue that learner engagement is subject to variable mediating factors inside and outside the language classrooms, which include learners’ own psychological states, broader social and cultural contexts as well as peer and teacher relationships; these variables interact with each other in a complex dynamic system, and the combination of these factors have an impact on learners’ engagement in the language tasks. Besides incorporating social engagement into the previous three-dimension engagement construct, Li and Li (2022) also identify crucial factors impacting learner engagement in EFL classrooms, including the instructor presence, learner presence, learning environment, and learning content. The authors warn that learner engagement is complex and inter-connected. These factors do not function in isolation and may jointly influence learners’ engagement in the context-specific flipped EFL classroom.

## Evaluating the impact of flipped classrooms on English for foreign language learners’ engagement

Given that there has been a growing number of research on engagement in EFL contexts, we analyze four papers to analyze the effectiveness of flipped instruction on EFL learners’ engagement. These papers are all related to learner engagement in flipped EFL settings, including China, South Korea, Iran and Vietnam (see Appendix Table 1).

Li and Li (2022) examined the effects of flipped instruction on the behavioral, emotional, cognitive and social engagement of EFL learners in a Chinese university context through an experimental design that involved 34 students in the flipped classroom (the experimental group) and 35 students in the non-flipped classroom (the control group). The results indicated that after 8 weeks of flipped instruction in the listening and speaking class, learners in the flipped class had higher levels of behavioral, cognitive and social engagement than those in the non-flipped class. In contrast, the difference in emotional engagement between the two groups was not statistically significant. Further, the authors identified four positive factors affecting learner engagement in the flipped EFL environment, alongside a range of negative elements such as learners’ lack of preparation, extra workload, technical problems and video qualities. The study exposed mixed results of flipped EFL classrooms, indicating that flipped instruction does not necessarily positively impact learners. The authors called upon instructors to beware of contextual complexity and sensitivity of flipped instruction.

Amiryousefi (2019) used mixed methods to investigate the impact of flipped instruction on 69 Iranian university students. Comparing the traditional and flipped listening and speaking classrooms, he found that learners were more engaged with course materials and learning content in the flipped classroom than those in the traditional class; learners in the flipped class also demonstrated more interest, confidence, and willingness to communicate and spent more time learning English than those in the traditional classroom. Although Amiryousefi did not classify learner engagement into various dimensions, we would argue that based on Svalberg’s language engagement definition mentioned earlier, learners taught in flipped classrooms had more robust behavioral, emotional and social engagement than those in traditional classrooms.

Lee and Wallace (2018) covered learner engagement in more detail by investigating 39 Korean EFL students in tertiary education. Through a mixed-methods research design, the author found that learners in the flipped classroom were more engaged with their learning than those in the non-flipped classroom. Specifically, students in the flipped classroom asked more questions in group discussions, appeared to be more invested in the learning process and tasks, and developed a deeper understanding of the learning content than those in the non-flipped classroom. On the other hand, the authors also mentioned that instructors implementing flipped instruction must reflect on learner engagement throughout the learning process, incorporate interactive activities during online tutorials in the pre-class phase, ensure the quality of videos and provide computers for learners at the school.

Unlike the three studies above, which prioritize the quantitative method over the qualitative part in the mixed-methods research design, Tran and Nguyen (2020) mainly adopted semi-structured interviews to understand how learners engage with the flipped English classroom in Vietnam. Interviews with ten students from the flipped classroom showed that in the behavioral engagement, students made great efforts to understand videos, complete quizzes before the class, and join interactive activities with great concentration. In emotional engagement, learners were confident and satisfied with their performance in classroom discussions because they were well-prepared; students were also in favor of the flexibility of flipped instruction as they could view pre-class materials at any time. In terms of cognitive engagement, students in the flipped classroom developed learning strategies such as using Zalo (a Vietnamese messaging app) to communicate before the class and watch the videos for general understanding and then for detailed information. However, the study also found that one student was concerned about his prior English proficiency and the distraction of digital gadgets.

## Discussion

The literature above reveals overall positive correlations between flipped classrooms and student engagement in the EFL learning context. This result is congruent with the study of Vitta and Al-Hoorie (2020), whose review of flipped language classrooms indicates flipped instruction generates favorable learning outcomes. The positive results of flipped learning in EFL contexts may be because learners are exposed to pre-class learning materials so that they are more prepared to participate in interactive-based language tasks (Lee and Wallace, 2018; Li and Li, 2022). Besides, the increased opportunities for interaction between instructor and learner may lead to closer teacher-student rapport and more peer trust (Vitta and Al-Hoorie, 2020).

However, the reviewed literature also shows some contradictory findings. For example, the emotional engagement of learners in the flipped and non-flipped classes in Li and Li’s (2022) study is not statistically different as opposed to the study of Amiryousefi (2019) and Tran and Nguyen (2020). In addition, in the research done by Lee and Wallace (2018), the successful implementation of flipped instruction hinges on the instructor, who is expected to provide immediate support to learners, conduct online tutorials before the class, and grant learners easy access to the Internet. One reason for contradictory findings may be that all the studies use relatively small samples, which prevent their results from being generalized. The inconsistency in the findings is also likely to be caused by the difference in teaching and learning contexts. This indicates that all the contextual factors must be considered before concluding that flipped instruction can significantly promote learner engagement. Turan and Akdag-Cimen (2019) echoed this view after reviewing forty-three articles about flipped EFL classrooms. The authors find that despite the generally positive results of most flipped EFL classes, some scholars report that several students express learning anxiety and nervousness due to the interactive nature of flipped learning and extra workload.

Therefore, the efficacy of the flipped classroom, particularly its effect on learner engagement, is context-specific and should be researched according to the local teaching and learning situation. The pedagogical implication is that if flipped instruction is applied to enhance learners’ engagement, restrictive factors that are likely to negate its effectiveness should be well noted. For example, instructors must pay attention to contextual elements, such as individual learner differences, learner preparedness, learning support and access to modern technology while designing and implementing flipped language courses (Li and Li, 2022).

## Conclusion

The accumulated research suggests that the flipped classroom has different definitions and its effects range from enhancing learning outcomes to developing learner engagement. However, despite generally favorable results, some findings in the reviewed articles also point toward a negative learning experience for some. Following this, more empirical research should be carried out in various EFL environments to measure learner engagement from multidimensional, holistic and dynamic perspectives. For example, what contribute to learner engagement in flipped learning environment, whether flipped classrooms can sustain learner engagement and how the individual engagement constructs change and interact over time remains largely underexplored. In addition, some of the reviewed papers do not have theoretical underpinnings. The dynamic motivation systems theory (Mercer and Dörnyei, 2020) and engagement with language (Svalberg, 2009, 2018) may offer promising theoretical foundations to study engagement in flipped language classrooms. In this way, we can gain more comprehensive knowledge about how multiple internal and external factors affect EFL learners’ engagement as well as how engagement constructs interact in the specific flipped EFL setting.

This mini-review article also has limitations. It only covers four critical studies associated with learner engagement in the flipped EFL contexts, which only provide a partial picture of the area of inquiry. Future researchers can conduct a systematic or scoping review on engagement in the flipped EFL contexts to investigate the effects of flipped instruction and the influencing variables in various teaching and learning environments.

## Author contributions

Both authors listed have made a substantial, direct, and intellectual contribution to the work, and approved it for publication.

## Conflict of Interest

The authors declare that the research was conducted in the absence of any commercial or financial relationships that could be construed as a potential conflict of interest.

## Publisher’s Note

All claims expressed in this article are solely those of the authors and do not necessarily represent those of their affiliated organizations, or those of the publisher, the editors and the reviewers. Any product that may be evaluated in this article, or claim that may be made by its manufacturer, is not guaranteed or endorsed by the publisher.

## Appendix

**APPENDIX TABLE A1:** A summary of major studies.

Study	Location	Subject area	Major findings
Li and Li, 2022	China	College English: Listening and Speaking	The flipped classroom improved learners’ behavioral, cognitive and social engagement, but not emotional engagement. Various factors were found responsible for learner engagement and disengagement.
Amiryousefi, 2019	Iran	College English: Listening and Speaking	The flipped classroom led to higher learner engagement with course materials and learning content, and learners were more interested, confident and willing to communicate in the flipped classroom than those in the non-flipped classroom.
Lee and Wallace, 2018	South Korea	College English (with a focus on academic speaking and writing)	Students in the flipped classroom were more actively engaged in classroom discussions, raised more questions, spent more time learning; and adopted more learning strategies than those in the non-flipped classroom. However, the flipped classroom also added to teachers’ workload.
Tran and Nguyen, 2020	Vietnam	College English (with a focus on tourism English)	Students had a robust engagement with the flipped classroom in the behavioral, emotional and cognitive dimensions. Nevertheless, one learner was worried about his prior English proficiency and complained about distraction problems caused by digital gadgets.
